# In Vitro Interaction of 5-Aminoorotic Acid and Its Gallium(III) Complex with Superoxide Radical, Generated by Two Model Systems

**DOI:** 10.3390/ijms21228862

**Published:** 2020-11-23

**Authors:** Lozan Todorov, Maria Traykova, Luciano Saso, Irena Kostova

**Affiliations:** 1 Department of Chemistry, Faculty of Pharmacy, Medical University—Sofia, 1000 Sofia, Bulgaria; irenakostova@yahoo.com; 2 Department of Physics and Biophysics, Faculty of Medicine, Medical University—Sofia, 1431 Sofia, Bulgaria; mlvtraykova@gmail.com; 3 Department of Physiology and Pharmacology “Vittorio Erspamer”, Faculty of Pharmacy and Medicine, Sapienza University, 00185 Rome, Italy; luciano.saso@uniroma1.it

**Keywords:** oxidative stress, gallium complex, 5-aminoorotic acid, antioxidant, superoxide radical, potassium superoxide, xanthine/xanthine oxidase

## Abstract

Increased levels of the superoxide radical are associated with oxidative damage to healthy tissues and with elimination of malignant cells in a living body. It is desirable that a chemotherapeutic combines pro-oxidant behavior around and inside tumors with antioxidant action near healthy cells. A complex consisting of a pro-oxidant cation and antioxidant ligands could be a potential anticancer agent. Ga(III) salts are known anticancer substances, and 5-aminoorotic acid (HAOA) is a ligand with antioxidant properties. The in vitro effects of HAOA and its complex with Ga(III) (gallium(III) 5-aminoorotate (GaAOA)) on the in vitro accumulation of superoxide and other free radicals were estimated. Model systems such as potassium superoxide (KO_2_), xanthine/xanthine oxidase (X/XO), and rat blood serum were utilized. Data suggested better antioxidant effect of GaAOA compared to HAOA. Evidently, all three ligands of GaAOA participated in the scavenging of superoxide. The effects in rat blood serum were more nuanced, considering the chemical and biochemical complexity of this model system. It was observed that the free-radical-scavenging action of both compounds investigated may be manifested via both hydrogen donation and electron transfer pathways. It was proposed that the radical-scavenging activities (RSAs) of HAOA and its complex with Ga(III) may be due to a complex process, depending on the concentration, and on the environment, nature, and size of the free radical. The electron transfer pathway was considered as more probable in comparison to hydrogen donation in the scavenging of superoxide by 5-aminoorotic acid and its gallium(III) complex.

## 1. Introduction

The superoxide radical (O_2_^•−^) is involved in many normal and pathological bio-reactions in the living body [1]. Extracellular superoxide is released by the cell types involved in the immune defense and many other types of cells [2,3,4]. Below concentrations of 10^−6^ M, superoxide stimulates cellular growth [2,5,6]. Within 1 × 10^−5^ and 3 × 10^−5^ M concentrations, it is involved in cellular growth arrest, apoptosis, and necrosis [1]. Through dismutation and following Fenton reaction, O_2_^•−^ produces the highly toxic hydroxyl (OH^•^) free radical. The latter is responsible for the formation of various reactive oxygen (ROS) and reactive nitrogen (RNS) species, involved in more or less advanced oxidative damage of lipids and tissues.

The persistent metabolic domination of the accumulation of free radicals over their elimination (named oxidative stress) compared to normal tissues is typical for cancer cells [1,7]. Reactive oxygen species (ROS) and oxidative stress (OS) play a significant role in carcinogenesis [8,9,10] and cancer treatment [11,12]. The subject is very complex and is being intensively investigated. Via a number of pathways, OS is involved in carcinogenesis, but it is also able to help eliminate malignant cells by altering their redox homeostasis. Metal-induced oxidative stress is known to cause a wide range of diseases—K. Jomova and M. Valko have explored the subject in detail with regard to several metals, normally occurring in the human organism [13]. In contrast, the very same phenomenon may be beneficial for patient health in certain clinical cases. Disruption of the redox homeostasis of malignant cells by way of metal complexes is an extensive field in cancer therapy research [2]. ROS are immunosuppressive agents in the cancer microenvironment, facilitating tumor invasion and metastasis [1,8]. Simultaneously, ROS and reactive nitrogen species inflict significant damage to cellular membranes via peroxidation of lipids and denaturation of biologically active proteins and amino acid residues, all this resulting in alteration of enzymatic activities and permeability of ion channels. Finally, these destructive processes lead to apoptosis or necrosis of the cells being attacked. To survive, the malignant cells keep the optimal concentrations of superoxide radical and hydrogen peroxide by expressing high activity of superoxide dismutase (SOD) and catalase (CAT) [14,15,16,17,18,19,20], this even leading to the development of chemotherapeutic resistance of the cancer cells [21].

The perfect chemotherapeutic must facilitate the O_2_^•−^ production and/or accumulation in the tumors’ environment while eliminating excessive superoxide radicals around healthy cells. This depresses the ROS and RNS accumulation around the cancer cells leading to their death while it increases the ROS and RNS around the normal cells, thereby stimulating their survival and proliferation. One possible approach to fulfill such requirements is the use of a complex consisting of a highly pro-oxidant cation surrounded by organic ligands with antioxidant properties. This complex has to be stable at normal, homeostatic pH, while decomposing at acidic pH typical for tumors.

Metallodrugs incorporating Ga(III) are being researched as promising anticancer agents [22,23], due to the strong similarities between the Ga(III) and the Fe(III) ions in terms of ionic radius, electronegativity, coordination geometry, electron affinity, and Lewis base affinity. Ga(III) has a stable valent state in physiological conditions, in contrast with Fe(III). Since cancer cells require iron in larger quantities, compared to normal cells [24], the introduction of Ga(III) in order to disrupt the iron-dependent metabolic pathways [25] in malignant cells seems a promising strategy in cancer treatment [26]. Ga(III) salts are well known anticancer agents [27,28]. Simple gallium salts such as gallium nitrate, gallium chloride, and gallium citrate have been investigated for their antitumor, anti-inflammatory, and antimicrobial properties [29]. Besides antineoplastic activity, gallium nitrate has been investigated as a treatment for elevated, cancer-related blood calcium levels [30]. Its effectiveness, when introduced to the body as a continuous infusion, has been comparable to—and in some cases is even better than—that of established antihypercalcemic drugs, such as calcitonin [31] and bisphosphonates [32,33]. Radioactive gallium is utilized as a diagnostic agent in cancer [34,35] and some other diseases [36,37]. A number of complexes of gallium have been researched in recent years for their biological activities [38]. The Ga(III) complex with 5-aminoorotic acid (HAOA) showed better in vitro antioxidant properties than the ligand alone [39,40]. The interaction of HAOA and gallium(III) 5-aminoorotate (GaAOA) with superoxide radical has still not been investigated. Generally, little investigation on the impact gallium substances have on oxidative stress has been carried out so far [28]. Increasing human exposure to that metal, resulting most prominently from technogenic pollution, makes that a subject of rising importance from the viewpoint of the authors of the present research paper. In this study, the in vitro interaction between a novel gallium(III) complex and an important member of the family of ROS (the superoxide radical ion) is investigated. The authors also present results from a number of additional experiments with the aim to elaborate on the probable mechanisms of that interaction.

In the present work, the in vitro interaction of GaAOA and HAOA was investigated using two model systems generating O_2_^•−^. The goal of the investigation was to observe if the compounds eliminate or help in the generation of superoxide in normal homeostatic pH of 7.45. In the presence of xanthine and xanthine oxidase (the X/XO system), the superoxide was a side product from the transformation of xanthine into uric acid (UA). Two parameters were measured in this model system, i.e., the luminol-dependent chemiluminescence (LDCL), dependent on the superoxide in the reaction medium, and the activity of xanthine oxidase (calculated by monitoring the formation of uric acid). The other model system consisted of potassium superoxide (KO_2_) solution in dehydrated dimethyl sulfoxide (DMSO) in K,Na-phosphate buffer (PBS, pH 7.45). In this medium, O_2_^•−^ was produced by the chemical transformation of KO_2_ into K_2_O. The superoxide formation was measured using LDCL alone. The effects of aqueous solutions of HAOA and GaAOA in concentration ranges of 10^−6^ to 3 × 10^−4^ M were estimated as percentage of the parameters measured for the model systems in the absence of these compounds. The ability to donate hydrogen and to participate in electron-transfer reactions of HAOA and GaAOA was estimated by measuring the radical-scavenging activities toward 2,2-diphenyl-1-picrylhydrazyl radical (DPPH^•^) and 2,2′-azino-bis(3-ethylbenzothiazoline-6-sulphonic free radical (ABTS^•+^).

## 2. Results

**Figure 1:**
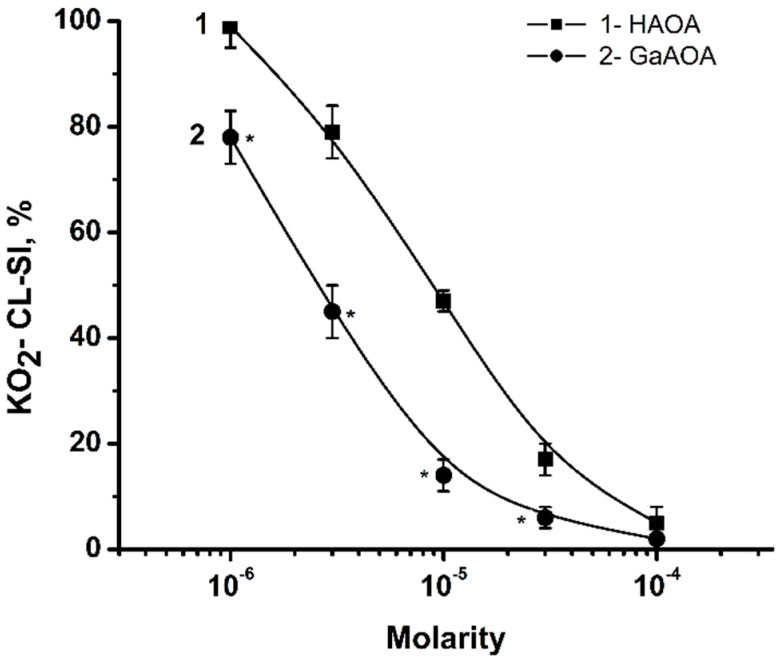
Effect of 5-aminoorotic acid (HAOA) (curve 1) and gallium(III) 5-aminoorotate (GaAOA) (curve 2) on luminol-dependent chemiluminescence (LDCL) in the presence of KO_2_-generated superoxide radical. Data = mean ± SD (*n* = 5). *, statistically significant difference from HAOA (*p* < 0.05).

The effect of 5-aminoorotic acid and its Ga(III) complex on the luminol-dependent chemiluminescence in the presence of KO_2_-generated superoxide radical is presented in Figure 1, presented as the chemiluminometric scavenging index (CL-SI, see Section 4.1). In the presence of both the ligand HAOA and complex GaAOA, LDCL decreased as concentrations increased. Within concentration limits of 10^−6^–3 × 10^−5^ M, both compounds acted as antioxidants. The IC_50_ values were 8.6 × 10^−6^ M and 3.8 × 10^−6^ M for 5-aminoorotic acid and its Ga(III) complex, respectively. Within the experimental error limits, at a concentration of 10^−6^ M HAOA did not show any interaction with O_2_^•−^ (Figure 1, curve 1), whereas GaAOA exhibited 80% CL-SI (Figure 1, curve 2).

Below 10^−4^ M at equimolar concentrations, the Ga(III) complex was a better scavenger of superoxide than 5-aminoorotic acid (Figure 1, curves 1 and 2). A comparison between curves 1 and 2 in Figure 1 revealed that the radical-scavenging activity (RSA) of the complex GaAOA corresponded to that of a threefold higher concentration of the ligand HAOA.

**Figure 2:**
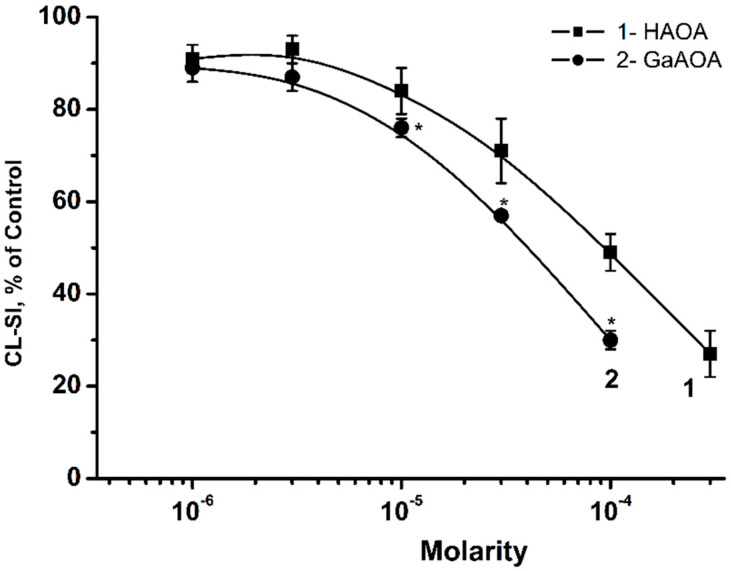
Effect of HAOA (curve 1) and GaAOA (curve 2) on the chemiluminometric scavenging index (CL-SI) in the superoxide-generating xanthine/xanthine oxidase (X/XO) model system. Data = mean ± SD (*n* = 5). *, statistically significant difference from HAOA (*p* < 0.05).

The effects of HAOA (curve 1) and GaAOA (curve 2) on the CL-SI in the presence of the superoxide-generating X/XO model system are illustrated in Figure 2. Within the experimental error limits, both compounds were scavengers of superoxide radical generated by the X/XO model system. The IC_50_ values observed were 2.92 × 10^−5^ M and 2.50 × 10^−5^ M for HAOA and GaAOA, respectively. Within concentrations of 3.0 × 10^−6^ M and 3.0 × 10^−4^ M, GaAOA (Figure 2, curve 2) exhibited better antioxidant activity compared to HAOA (Figure 2, curve 1).

**Figure 3:**
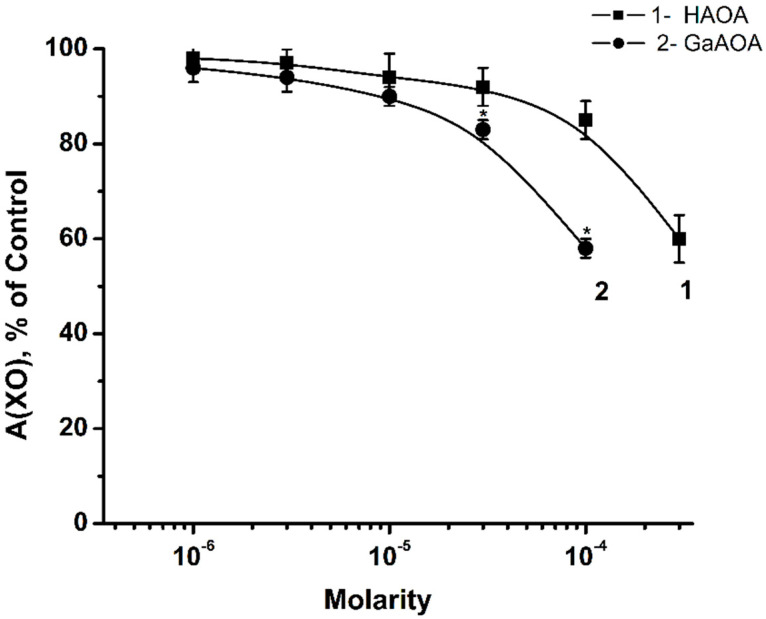
Effect of HAOA (curve 1) and GaAOA (curve 2) on the activity of the XO in the formation of uric acid (UA) from xanthine at pH of 7.45, according to UV detection of the uric acid. Data = mean ± SD (*n* = 5). *, statistically significant difference from HAOA (*p* < 0.05).

Above a concentration of 3.0 × 10^−6^ M, the CL-SI of GaAOA (Figure 2, curve 2) corresponded to that of a threefold higher concentration than that of HAOA (Figure 2, curve 1). By comparing Figure 1 and Figure 2, it was observed that the CL-SI of both compounds in the X/XO model system (Figure 2) were higher than those in the KO_2_ model system (Figure 1). This suggested lower antioxidant activity toward the X/XO-generated superoxide than toward KO_2_-generated O_2_^•−^. The reason for the lower antioxidant activities in the presence of the X/XO model system was found when calculating the activity of the enzyme in the presence of these compounds, the results of which are shown in Figure 3.

**Scheme 1:**
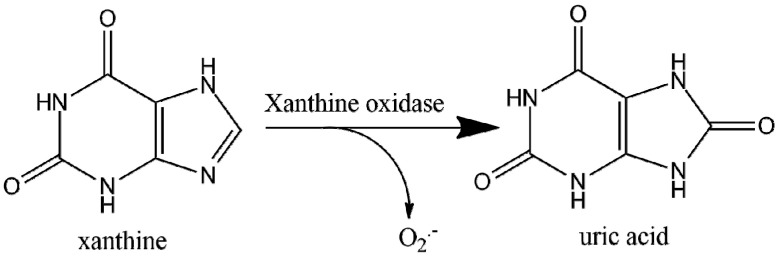
Production of uric acid and superoxide in the X/XO system.

In the xanthine/xanthine oxidase model system, the only source of superoxide was the reaction producing UA and superoxide (Scheme 1—see Section 4.3). Thus, the amount of uric acid corresponded to the amount of superoxide radicals formed during the enzymatic transformation of xanthine. Based on Figure 2 and Figure 3, it was proposed that the superoxide’s scavenging activities seen in Figure 2 followed the activities of xanthine oxidase shown in Figure 3. Data seen in Figure 3 indicated effects of the compounds investigated on the behavior of the X/XO model system. It was proposed that HAOA and GaAOA might interact either with the enzyme or with the substrate and that both actions would affect the formation of uric acid and superoxide.

**Figure 4:**
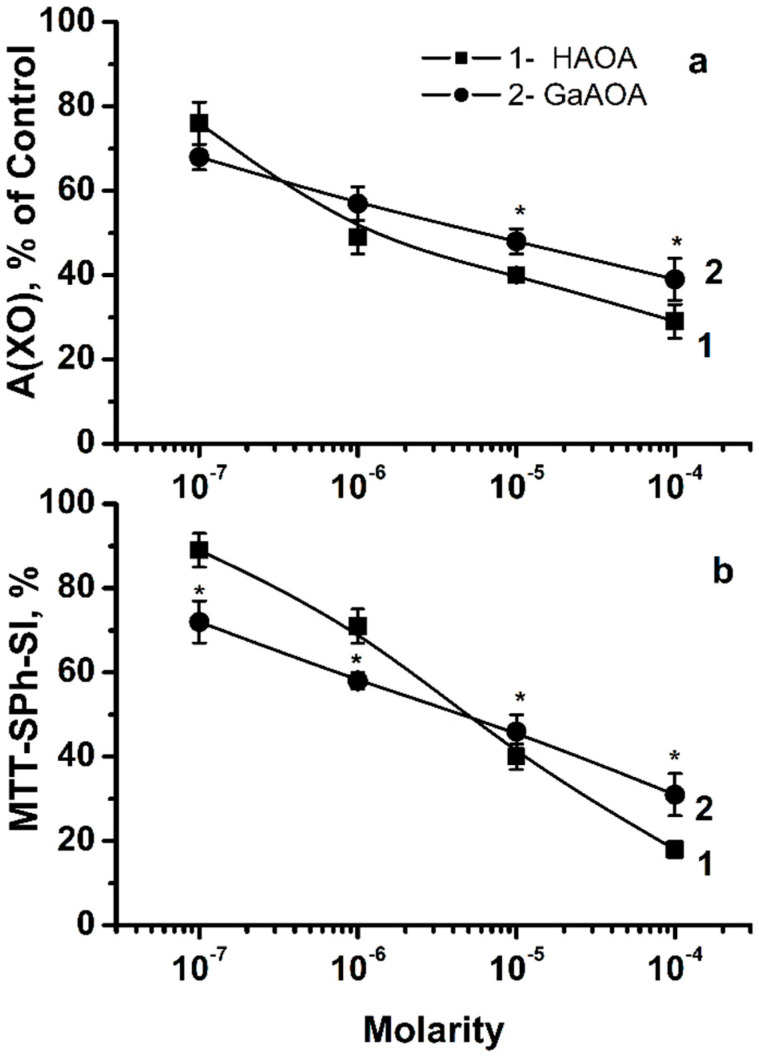
Effect of HAOA (curve 1) and GaAOA (curve 2) on (**a**) the activity of xanthine oxidase and (**b**) total free radicals’ formation in rat blood serum. Data = mean ± SD (*n* = 5). *, statistically significant difference from HAOA (*p* < 0.05).

The effects of HAOA and GaAOA on (a) the activity of XO and (b) total free radicals’ accumulation in rat blood serum are illustrated in Figure 4.

The concentration-dependent antioxidant effects of both compounds investigated are illustrated. At concentrations below 10^−6^ M, HAOA and GaAOA exhibited the same concentration-dependent decrease in the activity of XO (Figure 4a, curves 1 and 2). Above this concentration, HAOA decreased the XO activity to a slightly greater extent compared to GaAOA, this effect being very mild. Figure 4b shows that below 10^−5^ M GaAOA was a better scavenger of free radicals than HAOA at equimolar concentrations, whereas above this concentration HAOA was better than GaAOA.

**Figure 5:**
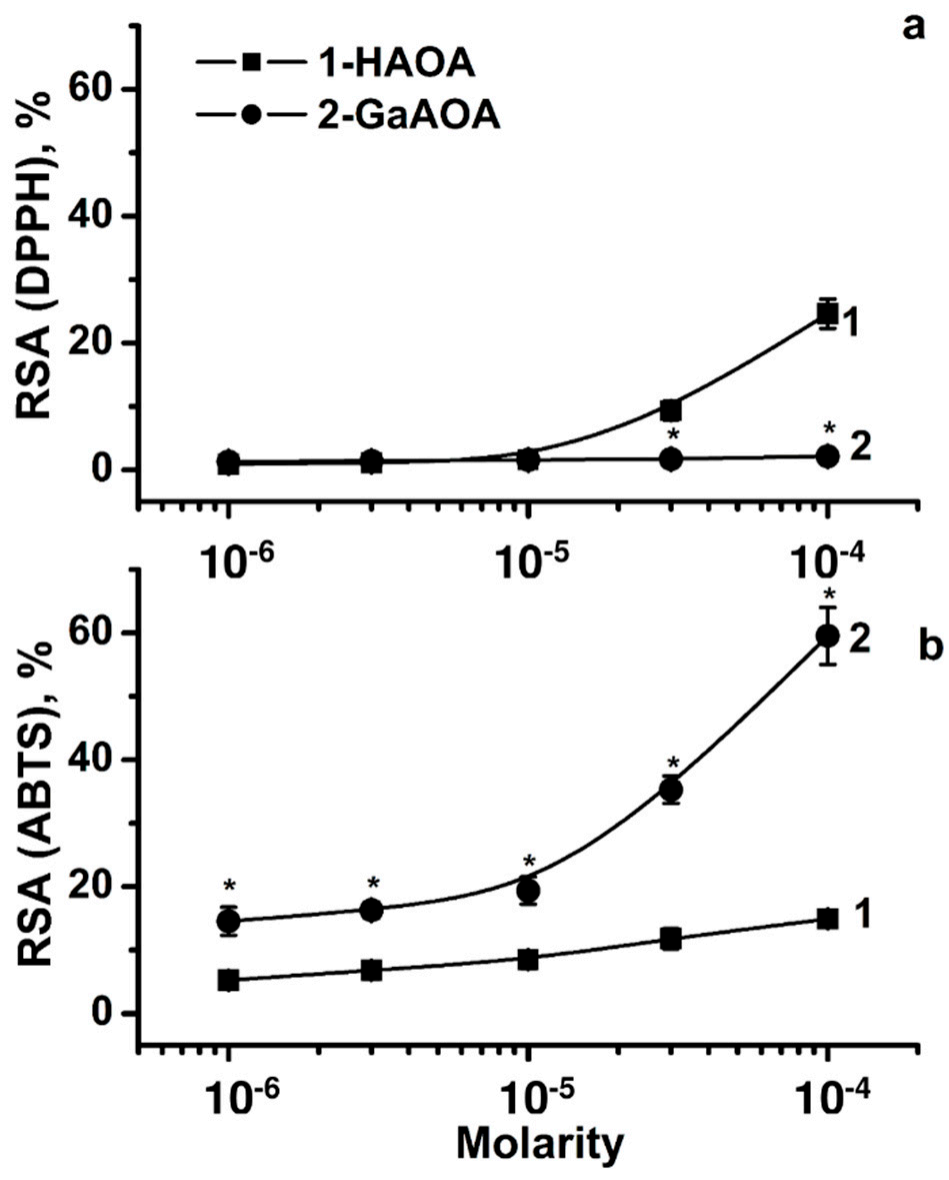
Radical-scavenging activities (RSAs) of HAOA (curves 1) and GaAOA (curves 2) toward (**a**) 2,2-diphenyl-1-picrylhydrazyl radical (DPPH^•^) and (**b**) 2,2′-azino-bis(3-ethylbenzothiazoline-6-sulphonic free radical (ABTS^•+^). Data = mean ± SD (*n* = 5). *, statistically significant difference from HAOA (*p* < 0.05).

The ability to donate hydrogen and to participate in electron transfer reactions of HAOA and GaAOA is shown in Figure 5.

The relative increase of RSA toward DPPH^•^ was modest below 10^−5^ M HAOA and more substantial above this concentration (Figure 5a, curve 1), with IC_50_ corresponding to 3.76 × 10^−5^ M. A very modest relative increase of RSA with concentrations of GaAOA was observed within concentrations between 10^−6^ M and 10^−4^ M, with IC_50_ found at 2.85 × 10^−5^ M (Figure 5a, curve 2). Within concentrations of 10^−6^ and 10^−4^ M, RSA of HAOA (Figure 5a, curve 1) increased from 2% to 25%, whereas that of GaAOA (Figure 5a, curve 2) increased from 1.3% to 2.12%. The increase of RSA toward ABTS^•+^ for HAOA was from 5% to 15% (Figure 5b, curve 1), whereas that of GaAOA increased from 14.5% to 59.95% (Figure 5b, curve 2). The IC_50_ values in Figure 5b correspond to 1.67 × 10^−5^ M and 3.08 × 10^−5^ M for HAOA and GaAOA, respectively. It was observed that the effect of GaAOA on RSA toward ABTS^•+^ was substantial, especially above 10^−5^ M solutions of the complex (Figure 5b, curve 2). In general, Figure 5a shows that HAOA was a better donor of hydrogen than GaAOA, while Figure 5b indicates a much better ability of the complex to participate in electron transfer reactions.

The IC_50_ trolox equivalents (TEs) of both investigated substances are presented in Table 1.

Data in Table 1 suggested that the elimination of free radicals in the presence of HAOA would be possible via both hydrogen donation and electron transfer, the former being more evident than the latter. In the presence of GaAOA, the electron transfer pathway was much more evident than the one involving hydrogen donation.

## 3. Discussion

Considering the role of superoxide in carcinogenesis [7,8,9,10,11], the interaction of an anticancer agent with this radical at normal homeostatic pH (7.45) is of great importance. If the compound prompts or accelerates the superoxide formation at normal homeostatic conditions, this increases the probability for malignization of healthy tissues. If the anticancer agent eliminates the O_2_^•−^ at pH of 7.45, this protects healthy cells from malignization. Ga(III)-containing compounds are promising anticancer agents [23,24,25,26,27,28,29,30,31,32,33,34], the metal ion being responsible for the toxicity against malignant cells [24,25,26,27]. The Ga(III) complex of 5-aminoorotic acid proved to be a better antioxidant than the ligand itself at normal homeostatic pH [39,40], but its interaction with O_2_^•−^ at this pH was not elucidated.

The LDCL experiments showed that, at concentrations above 10^−6^ M, both HAOA and GaAOA were scavengers of superoxide radicals (Figure 1 and Figure 2) in the KO_2_ and X/XO model systems. It was observed that, at equimolar concentrations, GaAOA was a better scavenger than HAOA. Figure 1 and Figure 2 indicate also that probably all organic ligands in GaAOA participated in scavenging of superoxide radicals. Figure 2 and Figure 3 indicate that, in the presence of the X/XO model system, the scavenging properties were interrelated with some interaction between scavenger and the components of the model system. It might be proposed that the in vitro antioxidant properties of HAOA and GaAOA in the X/XO environment were related to interactions with the components of this system. Figure 4 shows that both investigated compounds were antioxidants even in a more complicated environment such as rat blood serum. In this system, the production of superoxide was accompanied by the production and accumulation of a large variety of small and large free radicals [41]. In blood serum, both compounds acted as antioxidants toward X/XO (Figure 4a), their effects being influenced by interactions with all free radicals accumulated (Figure 4b). The relative differences between the effects of HAOA and GaAOA on the total free radicals’ accumulation (Figure 4b) could be related to fast and easy interactions with small radicals, whereas the interactions with large radicals were structurally hindered—an effect in agreement with previous observations [39]. It has been previously observed that the radical-scavenging effect of a substance is stronger for small and weaker for large free radicals [39]. Figure 5 indicates that both electron transfer and hydrogen donation might be involved in the antioxidant action of 5-aminoorotic acid and its gallium complex toward large free radicals. At concentrations below 10^−5^ M, both 5-aminoorotic acid and its complex with Ga(III) exhibited the same modest antioxidant action via hydrogen donation, whereas above this concentration HAOA was a better antioxidant than GaAOA through this pathway. GaAOA proved to be a much better radical scavenger via electron transfer reactions than HAOA.

The IC_50_ trolox equivalent calculations performed (Table 1) showed several tendencies:

- HAOA was a scavenger of both DPPH^•^ and ABTS^+•^ stable free radicals. Hydrogen donation seemed to be slightly more pronounced as an antioxidant mechanism of action compared to electron transfer. Both mechanisms could be possible, dependent on conditions in the medium.
- GaAOA demonstrated a much stronger tendency for participation in electron transfer reactions, compared to hydrogen donation.

The aforementioned observations were made with the consideration that both radicals are very large in size and complex in structure. Therefore, based on that data, conclusions could be drawn only regarding large, complex free radicals in an actual biological system.

Our investigation proved that both HAOA and GaAOA were scavengers of the superoxide radical at normal physiological pH (7.45). It suggested that in healthy tissues these compounds act as antioxidants, eliminating superoxide, thereby preventing oxidative cellular damage. The complexity of the content of the environment imposes a risk of various effects on the radical-scavenging activity of HAOA and GaAOA. Both 5-aminoorotic acid and its complex with Ga(III) may exhibit antioxidant action via hydrogen donation and electron transfer, the former being more probable in the presence of HAOA and the latter being more evident in the presence of GaAOA.

## 4. Materials and Methods

All materials and compounds were of finest grade (p.a.) purchased from Sigma-Aldrich (Sigma-Aldrich Chemie GmbH, Taufkirchen, Germany). Bi-distilled water was used for preparation of the solutions. The desirable concentrations of the compounds investigated were achieved by diluting the standard aqueous solution of concentrations of 10^−3^ M for HAOA and 3 × 10^−4^ M for GaAOA. The latter was the highest concentration possible achieved by dissolving GaAOA in water. In addition, 25.4 mU/mL xanthine oxidase were dissolved in 50 mM K, Na-phosphate buffer of pH = 7.45 (PBS) and used in the luminol-dependent chemiluminescence, and UV spectroscopic measurements. 5-Amino-2,3-dihydro-1,4phthalazinedione (luminol) was dissolved in small amount of 0.01 M NaOH, further diluted to 1.10–3 M in 50 mM K, Na-phosphate buffer of pH = 7.45, and pH was adjusted again to 7.45. Additionally, 1 mM KO_2_ solution in dehydrated DMSO was prepared directly prior to use. The 3 mM solution of xanthine was prepared by dissolving the compound in 0.1 N NaOH, and diluting with bi-distilled water. For the determination of the free radicals’ accumulation in the blood serum, 3 mg/mL aqueous solution of 3-(4,5-dimethyl-2-thiazolyl)-2,5-diphenyl-2H-tetrazolium bromide (MTT) was used.

The complex was synthesized by reaction of Ga(III) nitrate and the ligand, in amounts equal to the metal/ligand molar ratio of 1:3 using an earlier described procedure [42,43,44]. Reaction of Ga(III) and 5-aminoorotic acid afforded a complex that was found to be quite stable both in solid state and in solution. The preparation of the Ga(III) complex is summarized in the following equations representing the dissociation of HAOA and the respective interaction of AOA^−^ with Ga^3+^ ions:

HAOA ⇄ H^+^ + AOA^−^

[Ga(H_2_O)_n_]^3+^ + 3AOA^−^ ⇄ Ga^3+^(AOA^−^)_3_·H_2_O

where HAOA = C_5_N_3_O_4_H_5_ and AOA^−^ = C_5_N_3_O_4_H_4_^−^.

The new Ga(III) complex was characterized by elemental analysis, FT-IR, FT-Raman and UV–VIS spectroscopies. The used spectral analyses confirmed the composition and structure of the complex.

The binding mode of 5-aminoorotic acid to Ga(III) ions was elucidated by detailed vibrational analysis of theoretical and experimental IR and Raman spectra of the ligand and the complex. In the well-defined high-frequency field present in the IR and Raman spectra, extreme intensity changes were observed in going from the acid to the complex. In the spectral region of 3500–2000 cm^−1^, the O–H, N–H, and C–H stretches give rise to intense IR bands. The involvement of these groups in hydrogen bonds produces a relevant band broadening in the IR and Raman spectra. The double bond stretching vibrations ν(C=O) and ν(C=C) are the internal coordinates that dominate in the modes with fundamentals in the 1800–1600 cm^−1^ spectral range. One very strong band can be observed at 1691 cm^−1^ in the IR spectrum of the ligand assigned to the symmetrical stretching mode of C=O of the heterocyclic molecule. Opposite to the IR spectra, in this region of the Raman spectra, only a medium band at 1699 cm^−1^ for the free ligand was observed. These bands (broad and relatively strong in the IR spectrum) shifted in the spectra of the title complex. The same shifts were observed for the strong IR band at 1667 cm^−1^ tentatively assigned to the ν(C=O) mode of the carboxylic group and for the experimental Raman band at 1341 cm^−1^ assigned to the stretching ν(C–O) mode as a medium signal from the spectra of the free ligand. It must be mentioned that strong H-bonds are expected through the carboxylic groups. Different stretches of the uracil ring contributed to the bands in the 1600–900 cm^−1^ region slightly shifted in the spectra of the title complex. The metal affects the carboxylate anion as well as the ring structure. The spectra in the frequency region below 600 cm^−1^ are particularly interesting, since they provide information about the metal–ligand vibrations. The new bands in the 600–500 cm^−1^ region present only in the IR spectrum of the complex can be due to the Ga–O interactions. The Raman spectra are particularly useful in studying the metal–oxygen stretching vibrations, since these vibrations give rise to medium intensity bands in Raman, but are weak in the infrared spectra. The observed bands and their assignments are in accordance with the literature data for similar coordination compounds [44,45,46].

Luminol-dependent chemiluminescence was applied to estimate the radical-scavenging activity in the presence of the model systems containing KO_2_ and X/XO. A LUMAT LB9507 (Berthold Technologies GmbH & Co. KG, Bad Wildbad, Germany) apparatus was used for the LDCL investigations. The kinetics was measured with a delay time of 2 s, a measuring time of 3 s for a total measuring time of 600 s. The integral intensities for the first 10 s were used in data management.

The specific activity of XO in the X/XO model system was estimated by UV spectrophotometric measurement of the relative change of the characteristic signal of uric acid (UA) at 293 nm. This experiment was performed using a UV 1650PC Shimadzu spectrophotometer (Shimadzu, Duisburg, Germany). The delay time was 10 s, and the activity of the UA formation was computed by the program subroutine for the period 10–90 s. Independently, data for 600 s were collected.

Rat blood serum of a negative control rat was donated by the Department of Pharmacology and Toxicology, Medical Faculty of the Medical University-Sofia, Sofia, Bulgaria, as byproduct in their in vivo experiment. The amount of proteins was determined using the equation for proteins with contamination by unknown nucleic acids:

Proteins [mg/mL] = 1.55 × A_280_ − 0.76 × A_260_

where A_280_ and A_260_ are the absorptions of the characteristic wavelengths for the proteins and nucleic acids, respectively [45]. For the spectroscopic measurements, the amount of serum was adjusted to correspond to a concentration of proteins of 1 mg/mL.

The antioxidant capacity by way of hydrogen donation is often estimated by measuring the radical-scavenging activity (RSA) toward the stable 2,2-diphenyl-1-picrylhydrazy radical (DPPH^•^) [46,47,48], whereas participation in electron-exchange reactions may be tested by way of the ABTS^•+^ assay [49,50,51,52].

In order to compare the hydrogen-donating and electron-transfer properties of both investigated substances to an established standard, their IC_50_ trolox equivalents in the presence of DPPH^•^ and ABTS^•+^ were calculated. Standard solutions of Trolox (6-hydroxy-2,5,7,8-tetramethylchroman-2-carboxylic acid) were prepared in ethanol (DPPH^•^) and water (ABTS^•+^) at the following micromolar concentrations: 1, 5, 10, 20, 30, 40, 60, 100, 200, 300, 400, 600, 800, 1000, 1200, 1400, 1600, 1800, and 2000.

### 4.1. Assay for CL in the Presence of KO_2_

One mL of the control sample contained 0.05 mL KO_2_ solution, 0.05 mL luminol, and PBS. One mL of the sample volume contained 0.05 mL KO_2_, 0.05 mL luminol, 0.1 mL of the compound investigated in the desirable concentration, and PBS. The results were presented as chemiluminometric scavenging index (CL-SI) calculated as follows:

$$\mathrm{CL}-\mathrm{SI}=\frac{I_{\mathrm{sample}}}{I_{\mathrm{control}}}*100$$

where I_control_ and I_sample_ are the integral intensities measured for the KO_2_ alone and in the presence of the compound in a desirable concentration, respectively. The background measurement showed integral intensity of 10 and was subtracted from both control and sample measurements. For each compound at each desirable concentration, 5 parallel measurements were performed. Average values and standard deviations were used for further comparisons.

### 4.2. Assay for CL in the Presence of the X/XO Model System

One mL of the cuvette for the control measurement contained 0.02 mL XO solution, 0.1 mL X, 0.1 mL luminol, and PBS. One mL solution for the sample measurement contained 0.02 mL XO, 0.1 mL X, 0.1 mL of the compound investigated in the desirable concentration, and PBS. The CL-SI was determined using the same equation as the one shown in the KO_2_ assay. For each desirable concentration of the compounds investigated, 5 parallel measurements were performed. The average value and standard deviation were calculated and used in further comparisons.

### 4.3. Assay for UV Determination of XO Activity in the Presence of the X/XO Model System

One mL of the reaction mixture for the control measurement contained 0.02 mL XO, 0.1 mL xanthine, and PBS. One mL of the cuvette for the sample measurement contained 0.02 mL XO, 0.1 mL X, 0.1 mL of the compound investigated in a desirable concentration, and PBS. As no additional components in this model system were present, only uric acid (UA) and O_2_^•−^ were produced (Scheme 1):

As the final product of xanthine transformation was UA, the absorption at 293 nm (characteristic wavelength for UA) was measured for 10 min, using the molar extinction coefficient of 1.22 × 10^4^ M^−1^ cm^−1^ [53]. One unit of activity of XO was defined as the amount of the enzyme needed to convert 1 µmole of xanthine for 1 min in 1 mL reaction mixture at 298 K. Data for the activity of XO were presented as percentage of XO activity seen in the control measurement.

### 4.4. Assay for UV Determination of XO Activity in the Presence of Rat Blood Serum

One mL of the reaction mixture for the control measurement contained blood serum corresponding to 1 mg/mL protein content, 0.1 mL xanthine, and PBS. One mL of the cuvette for the sample measurement contained blood serum corresponding to 1 mg/mL protein content, 0.1 mL xanthine, the compound investigated in desirable concentration, and PBS. The activity of XO in the presence of a compound was presented as a percentage of the one seen for the control measurement.

### 4.5. Assay for Determination of Total Free Radicals’ Accumulation in Rat Blood Serum

One mL of the solution for the control measurement consisted of blood serum containing 1 mg proteins, 0.5 mL MTT, 0.1 mL X, and PBS. Additionally, 1 mL in the cuvette for the sample measurement contained the same components as the control plus the compound investigated in a desirable concentration. In the presence of free radicals, MTT transforms into MTT formazan, which has a characteristic absorption at 576 nm. The greater the content of free radicals in the solution, the higher is the absorption at 576 nm. The results were presented as SPh-SI.

### 4.6. RSA Assay with DPPH^•^

A standard solution of DPPH^•^ was prepared as was previously described [48]. The signal at 517 nm (characteristic band for DPPH^•^) was measured. The relative decrease in the absorption was monitored for 5 min after a 10 s lag time using the kinetic software of the apparatus. The absorption at 517 nm was recorded every 60 s.

Radical-scavenging activity, i.e., RSA (%), was calculated in the following way:

$$\mathrm{RSA}=\frac{A_{\mathrm{control}}-(A_{\mathrm{sample}}-A_{\mathrm{blank}})}{A_{\mathrm{control}}}*100$$

where A_control_, A_sample_, and A_blank_ represent the corresponding results for the control, sample, and blank measurement in both methods. The control measurement helped evaluate the absorbance of the characteristic signal of the stable free radical itself in the medium. The blank measurement accounted for the effect of the medium itself on the absorption at the radical’s characteristic wavelength, the radical being absent. Thus, (A_sample_ − A_blank_) demonstrates the diminishing of the characteristic absorption due to radical scavenging only. The higher the antioxidant activity of the investigated substance, the lower (A_sample_ − A_blank_) is compared to A_control_, therefore increasing RSA. Those data, along with the number of parallel measurements, were used in order to perform a statistical evaluation of relative differences between the RSAs of the varying substances at different concentrations.

The total volume of a solution in the cuvette was 2 mL. The composition of the samples is described in Table 2.

### 4.7. RSA Assay with ABTS^•+^

As prescribed by Erel [50], two solutions were prepared. Solution R1 is a medium of Na-acetate buffer with pH = 5.8. Solution R2 is composed of ABTS^•+^ dissolved in acetate buffer with pH = 3.8. The interaction of the investigated compounds with ABTS^•+^ was evaluated at 660 nm. This wavelength did not coincide with the characteristic bands of the tested substances. RSA was calculated using the same equation as for the DPPH method. The total volume of the solution in the cuvette was 1 mL. Five parallel blank, control, and sample measurements were used in order to calculate each RSA. Sample compositions are described in Table 3.

### 4.8. Trolox Equivalent Calculations

The interaction between the various trolox standard solutions and the stable radicals was investigated using the aforementioned DPPH^•^ and ABTS^•+^ assay methods (Section 4.7 and Section 4.8, respectively). The compositions of the trolox samples are described in Table 4 and Table 5 below.

For each trolox concentration, five parallel measurements were performed for the purposes of statistical evaluation. The RSAs were calculated as described previously (Section 4.6). The two RSAs closest to the calculated IC_50_ RSA values of HAOA and GaAOA were estimated.

For every tested concentration of each investigated substance, five parallel measurements were performed, each one representing one individual data point. Average values and standard deviations were calculated. Relative changes within the limits of experimental error were not discussed. Bartlett test was used to verify the significance in differences among standard deviations. The impact of the concentration of HAOA and GaAOA on the RSA of the solutions was statistically verified using one-way ANOVA, followed by Bonferroni post-test. The Bartlett test was used to verify that all standard deviations belonged to the same population. Differences with *p* < 0.05 were considered as statistically significant.

## 5. Conclusions

Based on the aforementioned experimental data, a number of probable conclusions can be made:

(1) 5-aminoorotic acid and its complex with Ga(III) are scavengers of superoxide radicals generated by different in vitro model systems at normal physiological pH (7.45). Both compounds may protect healthy tissues from oxidative cellular damage.
(2) All three organic ligands in the Ga(III) complex of 5-aminoorotic acid participated in the scavenging of O_2_^•−^.
(3) The scavenging activity of the compounds investigated toward superoxide radicals is affected by the presence of organic compounds and/or other types of free radicals in the environment.
(4) The electron transfer pathway was considered more probable than hydrogen donation in the scavenging of superoxide by the gallium(III) complex. 5-Aminoorotic acid seems to manifest its antioxidant action via both pathways.

The complex of gallium(III) and 5-aminoorotic acid combines the potential for anticancer activity (characteristic for Ga(III) ions) with pronounced antioxidant properties in vitro (characteristic for the molecule of 5-aminoorotic acid). The superoxide-scavenging properties of the complex (electron transfer) seem to manifest via a mechanism that differs from the ligand itself (both electron and hydrogen transfer). That observed difference may be due to a couple of factors:

(1) redistribution of electron charge densities in the ligands, resulting from their coordination with the gallium(III) ion;
(2) steric hindrance when it comes to the interaction between the relatively large DPPH radical and the antioxidant ligands, resulting in a semblance of a reduction in the proton-donating properties.

The present investigation helps establish a firm basis for additional research on the antioxidant properties of GaAOA and other novel gallium(III) complexes in general.

## Figures and Tables

**Table 1:** IC_50_ trolox equivalents (TEs) per milligram investigated substance (TE/mg) for HAOA and GaAOA. Higher values correspond to greater radical-scavenging activity.

Sample	DPPH^•^	ABTS^+•^
HAOA	474 ± 44	365 ± 37
GaAOA	17 ± 2	1698 ± 126

**Table 2:** Sample composition for the DPPH^•^ assay.

Sample Content	Blank	Control	Sample
Investigated substance	0.02 mL	none	0.02 mL
Ethanol	1.98 mL	none	none
DPPH^•^ solution	none	1.98 mL	1.98 mL
Bi-distilled water	none	0.02 mL	none

**Table 3:** Sample composition for the ABTS^•+^ assay.

Sample Content	Blank	Control	Sample
Investigated substance	0.04 mL	none	0.04 mL
R1	0.96 mL	0.94 mL	0.94 mL
R2	none	0.02 mL	0.02 mL
Bi-distilled water	none	0.04 mL	none

**Table 4:** Sample composition for the trolox DPPH^•^ assay.

Sample Content	Blank	Control	Sample
Trolox	0.02 mL	none	0.02 mL
Ethanol	1.98 mL	none	none
DPPH^•^ solution	none	1.98 mL	1.98 mL
Bi-distilled water	none	0.02 mL	none

**Table 5:** Sample composition for the trolox ABTS^•+^ assay.

Sample Content	Blank	Control	Sample
Trolox	0.04 mL	none	0.04 mL
R1	0.96 mL	0.94 mL	0.94 mL
R2	none	0.02 mL	0.02 mL
Bi-distilled water	none	0.04 mL	none

## Abbreviations

CAT	Catalase
DMSO	Dimethyl sulfoxide
GaAOA	Gallium(III) 5-aminoorotate
HAOA	5-Aminoorotic acid
LDCL	Luminol-dependent chemiluminescence
OS	Oxidative stress
RNS	Reactive nitrogen species
ROS	Reactive oxygen species
RSA	Radical-scavenging activity
SOD	Superoxide dismutase
UA	Uric acid
X/XO	Xanthine/xanthine oxidase

## References

[1] Day R.M., Suzuki Y.J. (2005). Cell proliferation, reactive oxygen and cellular glutathione. Dose-Response.

[2] Murrell G.A., Francis M.J., Bromley L. (1990). Modulation of fibroblast proliferation by oxygen free radicals. Biochem. J..

[3] Weaning R.S., Wever R., Doos D. (1975). Quantitative aspects of the production of superoxide radicals by phagocytizing human granulocytes. J. Lab. Clin. Med..

[4] Matsubara T., Ziff M. (1986). Increased superoxide ion release from human endothelial cells in response to cytokines. J. Immunol..

[5] Burdon R.H. (1995). Superoxide and hydrogen peroxide in relation to mammalian cell proliferation. Free Radic. Biol. Med..

[6] Li P.F., Dietz R., von Harsdorf R. (1997). Differential effect of hydrogen peroxide and superoxide anion on apoptosis and proliferation of vascular smooth muscle cells. Accumulation.

[7] Del Rio L.A. (1992). Metabolism of oxygen radicals in peroxisomes and cellular implications. Free Radic. Biol. Med..

[8] Prasad S., Gupta S.C., Tyagi A.K. (2017). Reactive oxygen species (ROS) and cancer: Role of antioxidative nutraceuticals. Cancer Lett..

[9] Dayem A.A., Choi H.Y., Kim J.H., Cho S.G. (2010). Role of oxidative stress in stem, cancer, and cancer stem cells. Cancers.

[10] Tafani M., Sansone L., Limana F., Arcangeli T., De Santis E., Polese M., Fini M., Russo M.A. (2016). The interplay of reactive oxygen species, hypoxia, inflammation, and sirtuins in cancer initiation and progression. Oxid. Med. Cell. Longev..

[11] Markovic L., Zarkovic N., Saso L. (2017). Controversy about pharmacological modulation of Nrf2 for cancer therapy. Redox Biol..

[12] Jungwrith U., Cowol C.R., Keppler B.K., Hartinger C.G., Berger W., Heffer P. (2011). Anticancer activity of metal complexes: Involvement of redox processes. Antioxid. Redox Signal..

[13] Jomova K., Valko M. (2011). Advances in metal-induced oxidative stress and human disease. Toxicology.

[14] Grigolo B., Linsigoli G., Toneguzzi S., Mazzetti I., Facchini A. (1998). Copper/Zink superoxide dismutase expression by different human osteosarcoma cell lines. Anticancer Res..

[15] Jansen A.M., Bosman C.B., Kuidenier L., Griffonen G., Lamers C.B., van Kreeken J.H., van de Velde C.J., Verspaget H.W. (1999). Superoxide dismutases in the human colorectal cancer sequence. J. Cancer Res. Clin. Oncol..

[16] Palazotti B., Pane G., Colavitti R., De Leo M.E., Bedogni B., Borello S., Galeotti T. (1999). Increased growth capacity of cervical carcinoma cell overexpressing manganous superoxide dismutase. J. Cancer.

[17] Malafa M., Margenthaker J., Webb B., Neitzel L., Christiphersen M. (2000). Mn DOS expression is increased im metastatic gastric cancer. J. Surg. Res..

[18] Yang J.Q., Li S., Huang Y., Zhang H.J., Dommann F.E., Buettner G.R., Oberley L.W. (2001). V-Ha-Rass overecpression induces superoxide overproduction and alters levels of primary antioxidant enzymes. Antioxid. Redox Signal..

[19] Chung-ma Ho J., Zheng S., Comhaer S.A., Farver C., Erzurum C.S. (2001). Differential expression of manganese superoxide dismutase and catalase in lung cancer. Cancer Res..

[20] Hur G.C., Cho S.J., Kim C.H., Kim M.K., Bae S.I., Nam S.W., Park J.W., Kim W.H., Lee B.L. (2003). Manganese superoxide dismutase expression correlates with chemosensitivity in human gastric cancer cell lines. Clin. Cancer Res..

[21] Suresh A., Guedez L., Moreb J., Zucali J. (2003). Overexpression of manganese superoxide dismutase promotes survival in cell lines after doxorubicin treatment. Br. J. Haematol..

[22] Ellahioui Y., Parashar S., Gómez-Ruiz S. (2017). Anticancer Applications and Recent Investigations of Metallodrugs Based on Gallium, Tin and Titanium. Inorganics.

[23] Chitambar C.R. (2012). Gallium-containing anticancer compounds. Future Med. Chem..

[24] Chitambar C.R., Antholine W.E. (2012). Iron-targeting antitumor activity of gallium compounds and novel insights into triapine^®^-metal complexes. Antioxid. Redox Signal..

[25] Chitambar C.R., Seligman P.A. (1986). Effects of different transferrin forms on transferrin receptor expression, iron uptake and cellular proliferation of human leukemic HL60 cells: Mechanisms responsible for the specific cytotoxicity of transferrin-gallium. J. Clin. Investig..

[26] Foster B.J., Clagett-Carr K., Hoth D., Leyland-Jones B. (1988). Gallium nitrate: The second metal with clinical activity. Cancer Treat. Rep..

[27] Valcheva-Traykova M., Saso L., Kostova I. (2014). Involvement of Lanthanides in the free radicals’ homeostasis. Curr. Top. Med. Chem..

[28] Todorov L., Kostova I., Traykova M. (2019). Lanthanum, Gallium and their impact on oxidative stress. Curr. Med. Chem..

[29] Chitambar C.R. (2010). Medical applications and toxicities of gallium compounds. Int. J. Environ. Res. Public Health.

[30] Warrell R.P., Bockman R.S., Coonley C.J., Isaacs M., Staszewski H. (1984). Gallium nitrate inhibits calcium resorption from bone and is effective treatment for cancer-related hypercalcemia. J. Clin. Investig..

[31] Warrell R.P., Israel R., Frisone M., Snyder T., Gaynor J.J., Bockman R.S. (1988). Gallium nitrate for acute treatment of cancer-related hypercalcemia a randomized, double-blind comparison to calcitonin. Ann. Intern. Med..

[32] Warrell R.P., Murphy W.K., Schulman P., O’Dwyer P.J., Heller G. (1991). A randomized double-blind study of gallium nitrate compared with etidronate for acute control of cancer-related hypercalcemia. J. Clin. Oncol..

[33] Cvitkovic F., Armand J.P., Tubiana-Hulin M., Rossi J.F., Warrell R.P. (2006). Randomized, double-blind, phase II trial of gallium nitrate compared with pamidronate for acute control of cancer-related hypercalcemia. Cancer J..

[34] Johnston G.S. (1981). Clinical applications of gallium in oncology. Int. J. Nucl. Med. Biol..

[35] Salloum E., Brandt D.S., Caride V.J., Cornelius E., Zelterman D., Schubert W., Mannino T., Cooper D.L. (1997). Gallium scans in the management of patients with Hodgkin’s disease: A study of 101 patients. J. Clin. Oncol..

[36] Raijmakers P.G.H.M., Groeneveld A.J., Teule G.J., Thijs L.G. (1996). Diagnostic value of the gallium-67 pulmonary leak index in pulmonary edema. J. Nucl. Med..

[37] Tehranzadeh J., Gubernick I., Blaha D. (1988). Prospective study of sequential technetium-99m phosphate and gallium imaging in painful hip prostheses (comparison of diagnostic modalities). Clin. Nucl. Med..

[38] Lessa J.A., Gabrieli L.P., Heloisa B. (2012). Gallium complexes as new promising metallodrug candidates. Inorg. Chim. Acta.

[39] Todorov L.T., Chifchiev B.B., Valcheva-Traykova M.L., Kostova I.P. (2018). Radical scavenging activity toward 2,2-diphenyl-1-picrylhydrazyl and hydroxyl radicals of 5 aminoorotic acid and its Ga(III) complex. Bulg. Chem. Commun..

[40] Todorov L., Valcheva-Traykova M., Traykov T., Kostova I. (2019). Impact of 5-aminoorotic acid and its complex with gallium(III) on the luminol-dependent chemiluminescence in presence of sodium hypochlorite. AIP Conf. Proc..

[41] Rokyta R., Stopk P., Holocok V., Krikava K., Pekarkova I. (2004). Direct measurement of free radicals in the brain cortex and the blood serum after nociceptive stimulation in rats. Neuro Endocrinol. Lett..

[42] Kostova I., Valcheva-Traykova M. (2015). New samarium(III) complex of 5-aminoorotic acid with antioxidant activity. Appl. Organomet. Chem..

[43] Kostova I., Valcheva-Traykova M. (2015). Synthesis, characterization, and antioxidant activity of a new Gd(III) complex. J. Coord. Chem..

[44] Kostova I., Valcheva-Traykova M., Balkansky S. (2016). Vibrational characterization and prooxidant activity of newly synthesized dysprosium(III) complex. J. Iran. Chem. Soc..

[45] Experimental Biosciences Quantifying Protein Using Absorbance at 280 nm Using a Formula for Possible Contaminations by Nucleic Acids. https://www.ruf.rice.edu/~bioslabs/methods/protein/abs280.html.

[46] Kedare S.B., Singh R.P. (2011). Genesis and development of DPPH method of antioxidant assay. J. Food Sci. Technol..

[47] Molyneux P. (2004). The use of the stable free radical diphenylpicrylhydrazyl (DPPH) for estimating antioxidant activity. J. Sci. Technol..

[48] Chrzczanowicz J., Gawron A., Zwolinska A., de Graft-Johnson J., Krajewski W., Krol M., Markowski J., Kostka T., Nowak D. (2008). α1 Antitrypsin. Clin. Chem. Lab. Med..

[49] Erel O. (2004). A Novel Automated Method to Measure Total Antioxidant Response against Potent Free Radical Reactions. Clin. Biochem..

[50] Erel O. (2004). A Novel Automated Direct Measurement Method for Total Antioxidant Capacity Using a New Gene-ration, more Stable ABTS Radical Cation. Clin. Biochem..

[51] Vural G., Gumusyayla S., Bektas H., Deniz O., Ergin M., Erel O. (2016). Dynamic Thiol-Disulphide Homeostasis in Patients with Multiple Sclerosis. World J Neurosci..

[52] Celic S., Baysal B., Sen S. (2019). Resveratrol attenuates benzo(a)pyrene- induced dysfunctions, oxidative stress and apoptosis in pancreatic beta-cells. Adv. Biosci. Biotechnol..

[53] Shintani H. (2013). Determination of xanthine oxidase. Pharm. Anal. Acta.

